# The Aggregate Index of Systemic Inflammation as a Predictor of Mortality in Stroke Patients

**DOI:** 10.7759/cureus.64007

**Published:** 2024-07-07

**Authors:** Adalet Göçmen, Tulin Gesoglu Demir

**Affiliations:** 1 Neurology, Sanliurfa Training and Research Hospital, Sanliurfa, TUR; 2 Neurology, Harran University Faculty of Medicine, Sanliurfa, TUR

**Keywords:** hemorrhagic, ischemic, mortality, the aggregate index of systemic inflammation, inflammation, stroke

## Abstract

Background and objectives

Stroke, a leading cause of mortality and disability, involves significant inflammation both before and after onset. This study investigates the relationship between the aggregate index of systemic inflammation (AISI) and mortality in stroke patients. The objective is to determine if AISI, an easily accessible biomarker, can predict stroke prognosis.

Materials and methods

In this retrospective study, the medical records of patients who presented to Harran University Neurology Clinic between January 2018 and September 2023 were reviewed. A total of 200 patients, 106 of whom were diagnosed as having an ischaemic stroke and 94 of whom were diagnosed as having a haemorrhagic stroke, were included in the study. A control group was also formed, which consisted of 100 people of similar age and sex with the patient group. The controls had neither chronic disease nor chronic drug use. Using biochemical and full blood count parameters, neutrophil-to lymphocyte ratio (NLR), platelet to lymphocyte ratio (PLR), lymphocyte to monocyte ratio (LMR), monocyte to lymphocyte ratio (MLR), neutrophil to high-density cholesterol ratio (NHR), monocyte to high-density cholesterol ratio, systemic immune inflammation index (SII), systematic immune response index (SIRI) and the AISI were calculated for all patients and the control group.

Results

A comparison of the two groups revealed significantly higher NLR, NHR, PLR, LMR, MLR, SII, SIRI and AISI values compared with the controls. NLR, PLR, SII, SIRI and AISI values were significantly higher in haemorrhagic stroke than in ischaemic stroke. Elevated NLR level and SII were correlated to mortality (respectively p:0.000, p = 0.017). SIRI (p = 0.189) and AISI (p = 0.162) were not correlated to mortality. However, receiver operating characteristic curve analysis determined that mortality increased for patients with AISI values above 507.45 (p = 0.003).

Conclusions

The AISI was found to be high among stroke patients, especially in haemorrhagic strokes. A relationship was observed between the increase in AISI above a certain value and mortality. The AISI is an accessible biomarker that shows inflammation in stroke patients. Therefore, it can be used to predict the prognosis of stroke.

## Introduction

Stroke is a cerebrovascular disease with acute onset in which the blood supply to the brain is impaired. It develops as a result of the occlusion of intracranial vascular structures or bleeding [1]. Of all stroke cases, 80% are ischemic and 20% are haemorrhagic [2]. Being sudden and unexpected, stroke causes devastation and is an important cause of mortality and disability [3].

Inflammation has an important role in the pathophysiology of stroke both before and after the stroke incident. Inflammation on the vessel wall resulting from an abnormal immune response forms a basis for atherosclerotic plaque, one of the precursors of ischemic stroke [4]. Damage-associated molecular patterns (DMAPs) are held responsible for post-stroke inflammation [5]. It aggravates blood-brain barrier injury, microvascular failure, brain oedema, and oxidative stress, triggering secondary brain injury resulting in neuronal death [6]. The process starts within minutes, increases in intensity in and around the lesion and may continue for weeks. It is observed in both types of stroke [7]. Therefore, it is thought that targeting inflammation in stroke therapy may be effective [8].

Blood biomarkers are widely used for disorders because they are easily accessible in most healthcare facilities and have low cost. They can guide the diagnosis and prognosis of disease. Many biomarkers have also been defined for inflammation. Among these, neutrophil-to-lymphocyte ratio (NLR), platelet-to-lymphocyte ratio (PLR), lymphocyte-to-monocyte ratio (LMR), neutrophil-to-high-density cholesterol ratio (NHR), monocyte-to-high-density cholesterol ratio (MHR), red blood cell distribution width (RDW), systemic immune inflammation index (SII) and systemic immune response index (SIRI) have been studied with respect to their relationship with stroke [9-15]. Increases in these indexes have been found to be correlated to stroke severity, increased mortality, sepsis risk and worsening functional outcomes. The aggregate index of systemic inflammation index (AISI), a novel biomarker, is calculated using four types of blood cells taking part in inflammation (neutrophils, monocytes, thrombocytes and lymphocytes). It has been studied among patients with coronavirus disease (COVID-19), hypertension and idiopathic pulmonary fibrosis and shown to correlate with these disorders and their prognoses [16-18]. In this study, we aimed to determine the presence of a relationship between the AISI and mortality in stroke.

## Materials and methods

Study design

In this retrospective study, the medical records of patients who presented to Harran University Neurology Clinic between January 2018 and September 2023 were reviewed. The study enrolled 200 patients older than 18 years of age who were diagnosed with ischemic or haemorrhagic stroke (where the aetiology did not involve trauma or vascular malformation) who presented to the hospital no later than 24 hours after the onset of neurological symptoms and were hospitalised. The control group was selected from patients who were admitted to the neurology outpatient clinic with any complaint, had no neurologic disease as a result of the examinations, were of similar age and gender to the patient group, and had no chronic systemic disease or drug use.

Ethical considerations

Ethics committee approval was obtained from the Harran University Faculty of Medicine Ethics Committee (HRÜ/23.23.38).

Study criteria

The inclusion criteria for the study were (1) patients older than 18 years, (2) diagnosed with ischemic or haemorrhagic stroke, (3) presenting to the hospital no later than 24 hours after the onset of neurological symptoms, and (4) hospitalised. The exclusion criteria included (1) patients with stroke caused by trauma or vascular malformation, and (2) patients with incomplete medical records.

Procedure

The medical records of the patients were retrospectively accessed from the hospital’s electronic file system. There were 106 patients with ischaemic stroke and 94 patients with haemorrhagic stroke. These patients’ age, sex, chronic disorders (atrial fibrillation, hypertension, diabetes, coronary artery disease, peripheral vascular disease, chronic renal failure, chronic liver disease, obstructive pulmonary disease) and medications were recorded. The patients’ laboratory tests ordered at first admission were collected. These results consisted of glucose, blood urea nitrogen, high-density cholesterol (HDL), low-density cholesterol (LDL), white blood cell count (WBC), lymphocyte count, neutrophil count, monocyte count, haemoglobin, platelet count, and RDW.

Using biochemical and full blood count parameters, NLR, PLR, LMR, MLR, NHR, MHR, SII, SIRI, and AISI were calculated for all patients and the control group.

Sample size calculation

The sample size for the study was determined based on the number of patients who met the inclusion criteria within the specified time frame (January 2018 to September 2023). A total of 200 patients were included in the study: 106 with ischaemic stroke and 94 with haemorrhagic stroke. The control group was selected to match the patient group in terms of age and gender distribution, with patients having no neurologic disease and no chronic systemic disease or drug use.

Statistical analysis

All study data were analysed using Statistical Package for the Social Sciences (IBM SPSS Statistics for Windows, IBM Corp., Version 20.0, Armonk, NY) software. Normally distributed quantitative variables of the two groups (stroke and control groups) were compared using Student’s t-test, while the variables without normal distribution were compared using the Mann-Whitney U test. The chi-squared test was used for qualitative variables. Numerical data were analysed by the Pearson correlation test. Quantitative variables are expressed as mean ± standard deviation and numerical values as number (%). A receiver operating characteristic (ROC) curve analysis was used to determine the predictive values of AISI for determining in-hospital mortality. Statistical significance was accepted where p < 0.05.

## Results

This study enrolled 200 patients diagnosed with stroke and 100 healthy control subjects. The demographic and clinical properties of the patient and control groups are shown in Table 1.

**Table 1:** Clinical-demographic properties of the patient and control groups Data are shown as mean (%) and standard deviation (±).

Clinical-demographic property	Patients n (%)	Controls n (%)
Age (years)	65.59 ± 12.41	65.04 ± 9.47
Female	95 (47.5%)	50 (50%)
Male	105 (52.5%)	50 (50%)
Ischaemic stroke	106 (53%)	_
Haemorrhagic stroke	94 (47%)	_
Hypertension	154 (77%)	_
Diabetes mellitus	67 (33.5%)	_
Atrial fibrillation	17 (8.5%)	_
Coronary artery disease	39 (19.5%)	_
Chronic obstructive pulmonary disease	4 (2%)	_
Peripheral vascular disease	1 (0.5%)	_
Chronic renal failure	19 (9.5%)	_
Medications		_
Antihypertensive	142 (71%)	_
Antidiabetic	58 (29%)	_
Acetylsalicylic acid	23 (11.5%)	_
Clopidogrel	6 (3%)	_
Warfarin sodium	12 (6%)	_
Rivaroxaban	1 (0.5%)	_
Statin	4 (2%)	_

The blood absolute leucocyte counts of the patient and control groups were compared. While the lymphocyte count was significantly lower in the patient group (p = 0.004), the neutrophil count was significantly higher in the patient group (p = 0.000).

The inflammation indexes of the patient and control groups are shown in Table 2. The comparison of the two groups revealed significantly higher NLR, NHR, PLR, LMR, MLR, SII, SIRI and AISI values in the patient group compared with the controls.

**Table 2:** Comparison of inflammation indexes of the patient and control groups Data are shown as mean (±) standard deviation. HDL: high-density cholesterol; MHR: monocyte/HDL ratio; LHR: lymphocyte/HDL ratio; NHR: neutrophil/HDL ratio; NLR: neutrophil/lymphocyte ratio; PLR: platelet/lymphocyte ratio; LMR: lymphocyte/monocyte ratio; MLR: monocyte/lymphocyte ratio; SII: systemic immune inflammation index; SIRI: systemic inflammation response index; AISI: aggregate index of systemic inflammation

Inflammation index	Patients (n =200)	Controls (n =100)	p-value
MHR	0.01 ± 0.02	0.01 ± 0.01	0.168
LHR	0.07 ± 0.33	0.05 ± 0.03	0.428
NHR	0.11 ± 0.05	0.22 ± 0.21	0.000
NLR	6.54 ± 7.08	2.15 ± 0.87	0.000
PLR	188.65 ± 134.66	126.96 ± 49.36	0.000
LMR	3.55 ± 2.61	4.20 ± 1.87	0.000
MLR	0.43 ± 0.51	0.28 ± 0.12	0.000
SII	1793.00 ± 2113.82	578.08 ± 343.48	0.000
SIRI	3.98 ± 6.49	1.27 ± 0.76	0.000
AISI	1115.50 ± 2130.85	337.74 ± 244.04	0.000

The laboratory test results of the patients are shown in Table 3. The NLR, PLR, SII, SIRI and AISI values were significantly higher in haemorrhagic stroke than in ischaemic stroke.

**Table 3:** Comparison of laboratory test results and inflammation indexes of haemorrhagic stroke patients and ischaemic stroke patients Data are shown as mean (±) standard deviation. HDL: high-density cholesterol; MHR: monocyte/HDL ratio; LHR: lymphocyte/HDL ratio; NHR: neutrophil/HDL ratio; NLR: neutrophil/lymphocyte ratio; PLR: platelet/lymphocyte ratio; LMR: lymphocyte/monocyte ratio; MLR: monocyte/lymphocyte ratio; SII: systemic immune inflammation index; SIRI: systemic inflammation response index; AISI: aggregate index of systemic inflammation; WBC: white blood cell count

Laboratory test results and inflammation indexes	Ischaemic stroke (n =106)	Haemorrhagic stroke (n =94)	p-value
HDL	35.55 ± 9.71	42.08 ± 11.36	0.000
WBC	10.02 ± 3.47	12.38 ± 4.76	0.000
Neutrophil	0.22 ± 0.27	0.23 ± 0.13	0.824
Lymphocyte	2.09 ± 1.29	1.71 ± 0.84	0.016
Monocyte	0.62 ± 0.27	0.74 ± 1.25	0.372
Platelet	257.84 ± 96.57	281.28 ± 104.40	0.102
MHR	0.01 ± 0.01	0.01 ± 0.03	0.938
LHR	0.10 ± 0.46	0.04 ± 0.03	0.139
NHR	0.22 ± 0.27	0.23 ± 0.13	0.830
NLR	5.19 ± 5.62	8.07 ± 8.19	0.005
PLR	168.38 ± 115.51	211.51 ± 150.81	0.026
LMR	3.78 ± 3.05	3.28 ± 1.97	0.170
MLR	0.39 ± 0.29	0.48 ± 0.67	0.227
SII	1365.40 ± 1616.86	2275.20 ± 2483.16	0.003
SIRI	3.01 ± 3.03	5.08 ± 8.80	0.032
AISI	751.88 ± 855.96	1525.53 ± 2927.09	0.015

**Figure 1:**
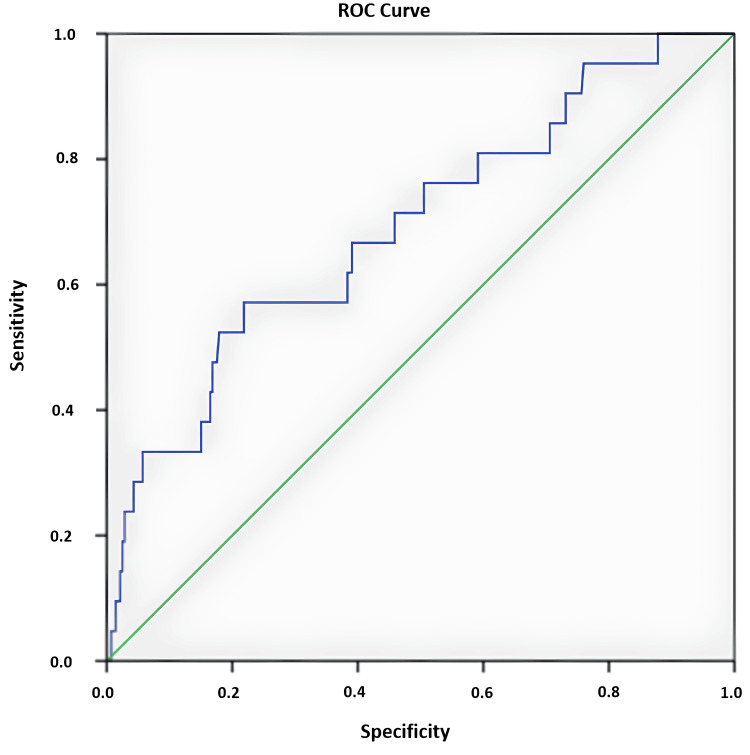
ROC analysis of AISI AISI: aggregate index of systemic inflammation; ROC: receiver operating characteristics curve

A total of 21 (10.5%) patients included in the study died during in-hospital follow-up; two (9.5%) of these were ischaemic stroke patients, and 19 (90.4%) were haemorrhagic stroke patients. Mortality was significantly higher in the haemorrhagic stroke group (p = 0.000). The mortality rate was correlated with elevated NLR (r = 0.214, p = 0.000) and SII (r = 0.217, p = 0.000). There was a weak correlation between SIRI (r = 0.134, p = 0.020), AISI (r = 0.135, p = 0.019) and mortality values. In the ROC analysis, it was determined that mortality increased for patients with AISI values ≥ 507.45 (Table 4 and Figure 1).

**Table 4:** Cut-off value for AISI AISI: aggregate index of systemic inflammation; AUC: area under the ROC curve

Risk factor	AUC (95% C.I.)	Cut Off	P	Sensitivity (%)	Specificity (%)
AISI	0.691 (0.568-0.815)	507.45	0.003	61.9	38.4

## Discussion

Inflammation plays a fundamental role in the pathogenesis of all stroke types from the onset of injury to the process of healing [10]. In this study, significantly higher inflammation indexes in the patient group compared to the control group indicate the importance of inflammation in stroke pathophysiology.

Previous studies have consistently shown that an increase in the count of leukocytes, which are part of inflammation, is associated with more severe disease and worse outcomes in stroke [19]. However, studies have found that a low lymphocyte count on admission increases the risk of infection and worsens the prognosis in patients with intracerebral haemorrhage [20]. In our study, consistent with the literature, leukocyte count among blood cells was significantly higher in the patient group compared to the control group. The count of lymphocytes in the patient group at the time of admission was lower than in the control group. Lymphocyte count was notably lower in haemorrhagic stroke than in ischaemic stroke but was not associated with mortality. Overall, leukocyte count is a crucial biomarker in stroke, providing insights into stroke severity, potential outcomes and treatment strategies.

There is accumulated evidence that inflammatory indexes calculated from leucocyte levels obtained in routine blood counts, such as neutrophils and lymphocytes, may provide valuable prognostic information in various disorders, including intracerebral haemorrhage and ischaemic stroke [21]. NLR, one of these indexes, comprehensively reflects the levels of neutrophils and lymphocytes and the balance between their different immunological activities. Various studies have supported the notion that NLR is an independent factor in predicting mortality, infarction volume, poor prognosis and recurrences of cerebral infarction among patients with ischaemic stroke [22]. In our study, NLR values were significantly higher in haemorrhagic stroke patients than in patients with ischaemic stroke and were associated with in-hospital mortality. There is a limited number of studies in the literature which compare NLR values in haemorrhagic and ischaemic stroke, and NLR values were found to be higher in patients with haemorrhagic stroke. Higher NLR values were linked to increased mortality in both ischaemic stroke and haemorrhagic stroke [23]. Studies have indicated that PLR has a decisive role in both functional outcomes in the first three months after stroke and the development of haemorrhagic transformation in ischaemic stroke patients with accompanying great artery stenosis [24]. In this study, we found that PLR values were higher with haemorrhagic stroke than with ischaemic stroke. These results suggest that NLR and PLR values can be used as biomarkers to predict the prognosis of stroke.

The SII and systemic inflammatory response index reflect both a strong pro-inflammatory response mediated by monocytes, neutrophils and thrombocytes and a lymphocyte-mediated anti-inflammatory response [25]. A study reported results suggesting elevated levels of these indexes may predict worse prognosis in ischaemic stroke and haemorrhagic stroke [26]. The scores of these indexes were higher in the patient group in our study. They were even higher in patients with haemorrhagic stroke than in those with ischaemic stroke. Higher SII values were correlated to in-hospital mortality.

The AISI is an inflammation index that was first studied in 2018 in relation to post-procedure prognosis for surgical patients and has been studied in many diseases since then [27]. A study on patients with idiopathic pulmonary fibrosis determined that it has a discriminatory power from healthy subjects and an ability to predict worse prognosis [28]. Our review of the literature suggested that this biomarker has not yet been studied in stroke patients. In our study, AISI values were higher in the patient group compared with the control group, and this is a finding that supports inflammation in the patient group. Also, it was higher in patients with haemorrhagic stroke than in patients with ischaemic stroke. This shows that inflammation is more prominent in haemorrhagic patients. We observed that mortality also increased as the AISI values increased, especially above 507.45. In light of these results, we can say that AISI can be used as a biomarker for predicting prognosis in stroke, especially haemorrhagic stroke, but more comprehensive studies are needed for this.

Our study, as well as previous studies, highlights the importance of inflammation in the pathophysiology of stroke. Therefore, targeting inflammation may have a crucial role in the treatment of stroke. We believe that studies on anti-inflammatory treatments should be carried out in the primary and secondary preventive treatment of stroke.

The limitations of the study include the fact that it was a retrospective study, the limited number of patients in the study due to some deficiencies in the hospital database, and the lack of an opinion on long-term disability and prognosis due to the retrospective nature of the study. In future studies, these limitations should be taken into consideration and prospective studies with large patient participation should be conducted to determine the effects of inflammation on long-term prognosis and disability.

## Conclusions

According to the results of this study, inflammation is prominent in stroke patients, especially in hemorrhagic stroke patients. The aggregate index of inflammation is a low-cost, easily accessible, practical and comprehensive index and gives us clues about the prognosis of stroke. Therefore, it can become a routinely used biomarker to determine the prognosis of stroke patients. We think that treatments targeting inflammation in stroke may be important in acute and secondary prevention. A wide range of prospective studies are needed to evaluate the the role of this biomarker in prognosis and to evaluate anti-inflammatory treatment in stroke.
